# A rare complication following excision and primary anastomosis urethroplasty: Osteomyelitis: a case report

**DOI:** 10.1097/MD.0000000000046757

**Published:** 2026-01-30

**Authors:** Ramazan Uğur, Çağri Şevik, Büşra Özcan Şevik, Halil Lutfi Canat

**Affiliations:** a Department of Urology, Başakşehir Çam and Sakura City Hospital, Istanbul, Turkey; b Department of Infectious Diseases, Başakşehir Çam and Sakura City Hospital, Istanbul, Turkey.

**Keywords:** infection, osteomyelitis, urethroplasty

## Abstract

**Rationale:**

Urethral stricture is the scarring and healing of the urethral epithelium or the corpus spongiosum layer just beneath it, following damage to the urethra for any reason. The curative and the gold-standard treatment is urethroplasty. The management of posterior urethral strictures is 1 of the most difficult problems for urologists and requires special attention and reconstructive experience.

**Patient concerns:**

A 79-year-old male who developed osteomyelitis following posterior urethroplasty.

**Diagnoses:**

Osteomyelitis was diagnosed after magnetic resonance imaging and bone biopsy. Histopathological examination confirmed osteomyelitis, and tissue cultures grew *Escherichia coli* and *Klebsiella* species.

**Interventions:**

The patient was initiated on intravenous meropenem therapy. Based on recommendations from clinical microbiology and infectious diseases specialists, a prolonged 6-week antibiotic regimen was administered.

**Outcomes:**

After parenteral antibiotic treatment, the patient’s clinical and laboratory parameters returned to normal.

**Lessons:**

To the best of our knowledge, this complication has not been previously described, making our case the first reported and, therefore, unique.

## 1. Introduction

Urethral stricture is a pathological condition characterized by fibrosis and narrowing of the urethral lumen due to scarring of the urethral epithelium or corpus spongiosum following injury from various causes. This process results in obstructive lower urinary tract symptoms that can significantly impact quality of life. The etiology includes idiopathic, iatrogenic, traumatic, and infectious causes. The choice of treatment is influenced by multiple factors, including the stricture’s location and length, the extent of fibrosis, the surgeon’s expertise, and the preferences of both the patient and the surgeon.^[1]^ Posterior urethral strictures primarily result from trauma-related urethral injuries associated with pelvic fractures, as well as iatrogenic causes involving the bladder neck, vesicourethral anastomosis, and membranous urethra.^[2,3]^ The complex anatomical structure of the posterior urethra, along with the challenges in accessing the stricture site in complicated cases, makes management difficult and necessitates expertise in reconstructive surgery.^[4,5]^ In this case report, we present a patient who developed osteomyelitis following posterior urethroplasty. The case is unique and contributes meaningfully to the urological literature, particularly in the context of complex posterior urethroplasty. Based on our literature review, we believe this to be the first reported case in the literature.

## 2. Case presentation

**Figure 1.**
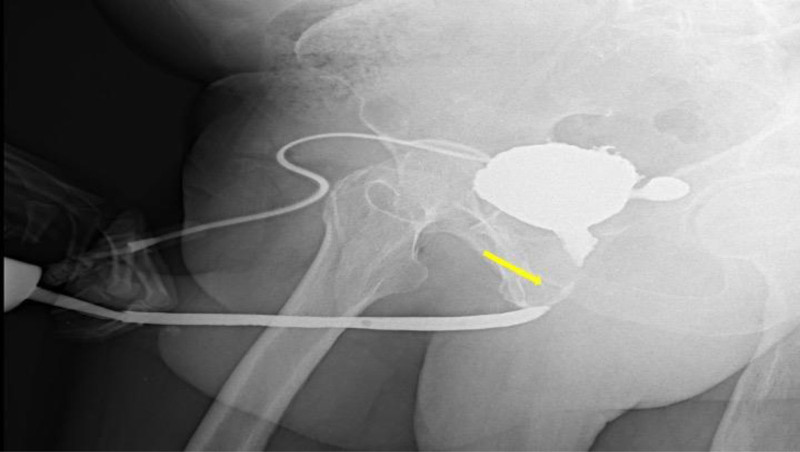
Preoperative retrograde urethrography. The yellow arrow indicates the site of urethral stricture.

The present case was diagnosed, managed, and followed between January 2024 and November 2024 at the Department of Urology, Başakşehir Çam and Sakura City Hospital. Written informed consent was obtained from the patient for the publication of this case report and any accompanying images. A 79-year-old male patient presented to our clinic with symptoms, including difficulty urinating, a sensation of incomplete bladder emptying, and post-micturition dribbling. His medical history included an open simple prostatectomy and prior endoscopic stone surgery. Uroflowmetry suggested a stricture pattern, prompting further evaluation with retrograde urethrography (RUG) and urethroscopy, which revealed an approximately 2.5-cm stricture in the bulbar-membranous urethra (Fig. 1). The patient underwent excision and primary anastomosis urethroplasty, corporal separation, and inferior pubectomy. Because of prior surgeries, the urethra was severely fibrotic and fragile. An approximately 2.5-cm stricture segment was excised. The integrity of the anastomosis was assessed using lubricant gel to check for extravasation, and after confirming the absence of leakage, the anastomosis was deemed secure.

After 3 days of inpatient follow-up, the surgical wound and overall condition were reassessed and the patient was discharged with both a urethral catheter and a suprapubic bladder catheter (SBC). On postoperative day 14, a pericatheter RUG was performed to evaluate for extravasation. No extravasation was detected, leading to the removal of the urethral catheter and clamping of the SBC. The patient was monitored for an additional day, during which no complications were observed, and the SBC was subsequently removed. Antibiotic therapy was continued while the catheter remained in place. Follow-up evaluations, including uroflowmetry and post-void residual urine assessment, were conducted at the 6th week and 3rd month postoperatively.

**Figure 2.**
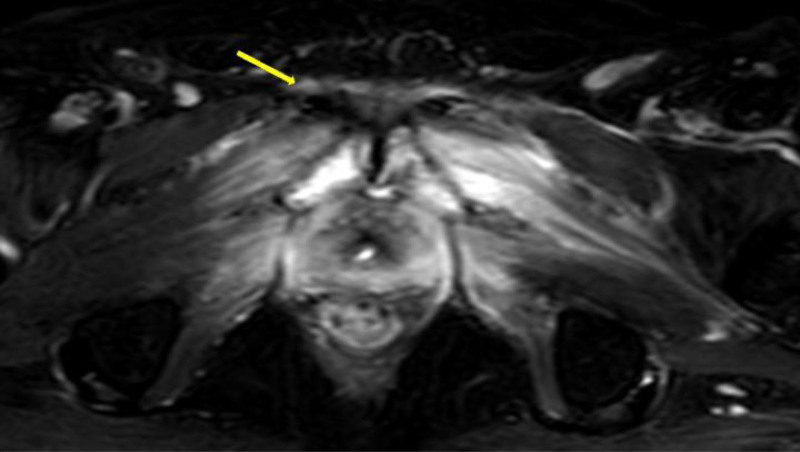
Contrast-enhanced pelvic magnetic resonance imaging. The yellow arrow indicates the infected area.

**Figure 3.**
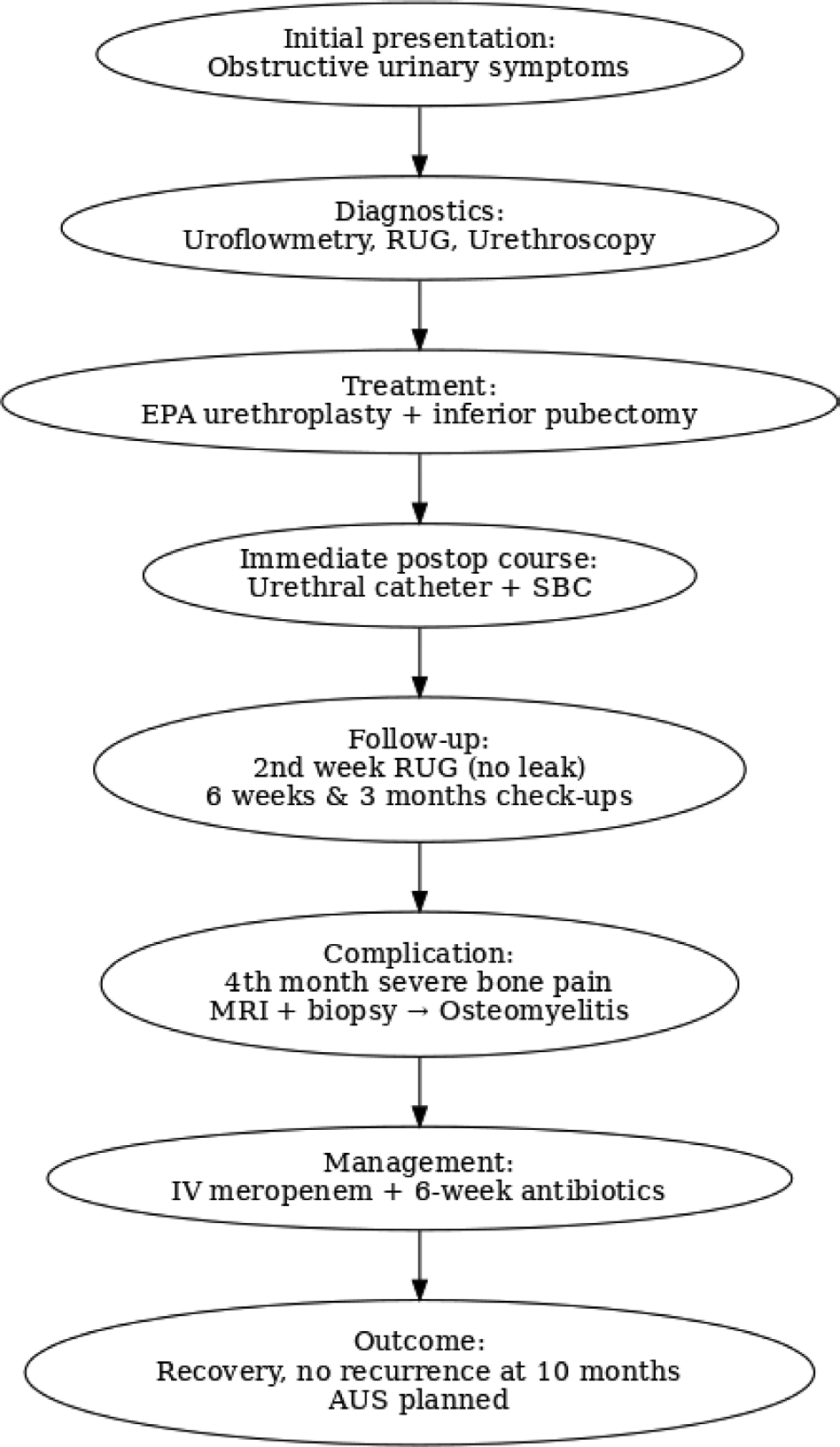
Flow chart showing the clinical course, diagnosis, treatment, complication, and outcome in the presented case. AUS = artificial urinary sphincter, EPA = excision and primary anastomosis, IV = intravenous, MRI = magnetic resonance imaging, RUG = retrograde urethrography, SUC = suprapubic bladder catheter.

During the patient’s 4th-month follow-up, he presented with severe hip and bone pain. Pelvic magnetic resonance imaging revealed contrast enhancement at the operative site, pelvic muscles, and the inferior pubic ramus at the level of the symphysis pubis (Fig. 2). Osteomyelitis was suspected, prompting a bone biopsy of the symphysis pubis, with simultaneous cultures sent for analysis. Histopathological examination confirmed osteomyelitis, and cultures grew *Escherichia coli* and *Klebsiella* species. The patient was initiated on intravenous meropenem therapy. Based on recommendations from clinical microbiology and infectious diseases specialists, a prolonged 6-week antibiotic regimen was administered. At the 10-month follow-up, no recurrence of stricture was observed. Following clinical stabilization, the placement of an artificial urinary sphincter is planned (Fig. 3). The patient expressed satisfaction with the treatment outcome despite the prolonged antibiotic therapy. He reported significant improvement in his quality of life and was grateful for the close follow-up that led to the timely diagnosis.

## 3. Discussion

Urethral strictures primarily impair patients’ quality of life; however, in some cases, they may lead to severe complications such as sepsis, which may be life threatening.^[5,6]^ Urethroplasty, recognized as the gold standard for urethral stricture treatment, is a technically demanding procedure with which many urologists have limited experience. Numerous studies in the literature report outcomes of urethroplasty performed with various techniques, including recurrence rates and associated complications. Studies on posterior urethroplasty have reported success rates ranging from 82% to 95%. Despite these high success rates, various complications have been documented, including restenosis, erectile dysfunction, incontinence, chordee, urethrocutaneous fistula, and position-related issues. Another 2 studies highlighted that the complete and thorough excision of scar tissue is the most critical factor for achieving success.^[7,8]^ In a study insufficient lateral fixation of the prostatic mucosa, anastomotic tension, and inadequate excision of scar tissue were identified as the most significant factors contributing to recurrent stricture formation.^[6]^

Complications related to the high lithotomy position during surgical procedures should be carefully considered. These may include neurapraxia, rhabdomyolysis, and lower extremity compartment syndrome. The length of the stricture, duration of the surgery, and positioning are significant factors contributing to these complications. Other less common complications of posterior urethroplasty include rectal injury, fistula formation, wound infection, perineal nerve injury, and recurrent urinary tract infections.^[1,3,8]^ Postoperative infectious complications following posterior urethroplasty most commonly include wound infections, perineal abscesses, and recurrent urinary tract infections, which are usually managed successfully with drainage and short-term antibiotics. The isolation of *E. coli* and *Klebsiella* species in our patient is consistent with previous reports of urinary tract pathogens in large clinical series. Furthermore, recent evidence highlights the role of bacterial virulence factors, such as chaperone-usher fimbriae and curli fimbriae, in the persistence and severity of infections caused by uropathogenic *E. coli*. These findings may provide a microbiological explanation for the unusual severity of postoperative infection in our case.^[9,10]^ Fistula formation and superficial wound breakdown have also been reported in complex cases. In contrast, osteomyelitis of the pubic bone has not been previously described as a complication of excision and primary anastomosis urethroplasty. Our case, therefore, highlights a unique and clinically significant infection that differs from the more common postoperative complications, emphasizing the importance of considering this rare entity in patients presenting with persistent bone pain and systemic signs of infection after urethroplasty. Especially in challenging urethroplasty cases involving inferior pubectomy, postoperative recurrent infections, bone and hip pain, and recurrent fever should prompt consideration of this complication, supported by radiological correlation, laboratory findings, and clinical signs. When this complication occurs, prompt management with appropriate pathogen-targeted antibiotic therapy, supportive care, and bed rest is essential. It should also be remembered that patients with a history of diabetes mellitus, immunosuppression, or medications that can cause these conditions, as well as those with a history of radiotherapy, may experience prolonged wound healing and maturation. Ensuring a watertight anastomosis during the operation and confirming the absence of extravasation with a pericatheter RUG postoperatively, followed by the removal of the urethral catheter after confirming no extravasation, and keeping the catheter in place for a longer duration than usual, are essential surgical principles to prevent such complications. As in our case, even when all necessary precautions are taken, it is crucial to remain alert to complications and to apply early diagnosis and treatment based on the clinical signs and findings.

## Author contributions

**Conceptualization:** Ramazan Uğur, Çağri Şevik.

**Data curation:** Ramazan Uğur, Çağri Şevik, Büşra Özcan Şevik.

**Formal analysis:** Ramazan Uğur.

**Funding acquisition:** Ramazan Uğur.

**Investigation:** Ramazan Uğur, Çağri Şevik.

**Methodology:** Ramazan Uğur.

**Project administration:** Ramazan Uğur.

**Resources:** Ramazan Uğur, Büşra Özcan Şevik.

**Software:** Ramazan Uğur.

**Supervision:** Ramazan Uğur, Halil Lutfi Canat.

**Validation:** Ramazan Uğur.

**Visualization:** Ramazan Uğur.

**Writing – original draft:** Ramazan Uğur, Çağri Şevik.

**Writing – review & editing:** Ramazan Uğur.
